# Ubiquinol in Fertility and Reproduction: A Conditionally Essential Nutrient for Critical Early-Life Stages

**DOI:** 10.3390/nu18010156

**Published:** 2026-01-02

**Authors:** Emma J. Derbyshire, Sergej M. Ostojic, Ahmed T. Alahmar

**Affiliations:** 1 Nutritional Insight, Epsom KT17 2AA, UK; 2 Department of Nutrition and Public Health, University of Agder, Universitetsveien 25, 4604 Kristiansand, Norway; sergej.ostojic@uia.no; 3 Department of Medical Physiology, College of Medicine, University of Babylon, Hilla 51001, Iraq; ahmed.t.alahmar@gmail.com

**Keywords:** advancing age, coenzyme Q10, conception, embryo development, fertility, infertility, oocyte quality, sperm function, ubiquinol

## Abstract

**Background/Objectives**: Infertility is a multifactorial condition with an etiopathology that remains largely unclear. Although substantial evidence implicates oxidative stress (OS) as a key contributor to both male and female infertility, targeted strategies for OS-mediated reproductive dysfunction are still not well defined and require further investigation. Ubiquinol is the reduced form of Coenzyme Q10 involved in mitochondrial bioenergetics. It can be synthesized by humans endogenously or provided by dietary sources—typically egg yolks, oily fish, organ meats, and in smaller amounts in nuts and seeds and leafy green vegetables. The present article reviews possible mechanisms through which Ubiquinol plays a role in the regulation of fertility and reproduction, discussing why it could be positioned as a conditionally essential nutrient. Several questions and areas for further inquiry are also proposed. **Methods**: The present position paper narratively summarizes evidence related to Ubiquinol fertility and reproduction, focusing on the literature from PubMed, Science Direct, and Semantic Scholar. **Results**: Research advancements suggest that when physiological demands rise during certain life stages, e.g., the reproductive years, the amount of Ubiquinol produced internally may not be enough to meet heightened needs, particularly with advanced maternal/paternal age. This places a heavier reliance on obtaining Ubiquinol from the diet, thus presenting itself as a conditionally essential nutrient during certain life stages. **Conclusions**: Overall, Ubiquinol appears to enhance mitochondrial energy production and antioxidant defense in gametes, a process that appears to aid sperm function, oocyte quality, and early embryo development. Collectively, these data indicate a key physiological role for Ubiquinol in male and female fertility, especially given its age-related decline.

## 1. Introduction

Reproduction is a physiologically costly process that uses considerable amounts of energy [1]. Ubiquinol, the reduced form of Coenzyme Q10 (CoQ10), forms a key part of the Ubiquinone–Ubiquinol cycle within mitochondria and is central to how cells, including reproductive cells, make energy [2]. It is the only lipid soluble antioxidant synthesized by human cells and the predominant form (>99%) of CoQ10 in the human body [3]. Female mature oocytes house around 100,000 mitochondria compared with around 1000–2500 mitochondria in other human cells [4,5]. Similarly, sperm mitochondria produce energy for the movement of the sperm, with a mature sex cell containing approximately 50–75 mitochondria in its midpiece [6,7]. Many stages of gametogenesis (sperm and oocyte maturation) are also sensitive to OS [8], and Ubiquinol as an antioxidant may help to attenuate this [9].

In normal, healthy conditions, the body typically produces enough Ubiquinol to meet cellular energy and antioxidant needs [2]. However, in certain circumstances where there may be higher metabolic and physiological demands, e.g., genetic factors, chronic diseases, mitochondrial dysfunction, or aging processes, the involved Ubiquinol production may be insufficient [2,10]. In modern society, infertility affects approximately 20–30% of women of reproductive age, and male factors are implicated in up to 50% of infertility cases, with nutrition and lifestyle increasingly recognized as influential contributors [11].CoQ10, particularly in its reduced form, Ubiquinol, is increasingly being recognized for its potential role in fertility health [12]. This review explores the latest scientific evidence supporting the potential role of Ubiquinol as a conditionally essential nutrient during life stages related to fertility, infertility, and reproductive health.

## 2. Essential or Conditionally Essential?

It is well recognized that our diets provide a range of essential nutrients and non-essential compounds [13]. In general terms, essential nutrients are identified when their removal contributes to a state of metabolic and/or clinical hypofunction and/or failure to grow, which could be reversed if that nutrient was introduced back into the diet [13,14]. In Europe, the European Food Safety Authority (EFSA) simply defines an essential nutrient as “*any substance which a living organism must consume from the diet in order to support normal health, development and growth*” [15].

Conditionally essential nutrients are those that the body can typically synthesized in adequate amounts under normal conditions, but during specific physiological states or stressors—such as illness, rapid growth, or metabolic imbalance—endogenous production may become insufficient, making dietary intake necessary to maintain optimal function [16]. In this context, arginine, carnitine, choline, creatine, cysteine, glycine, and taurine are all considered conditionally essential nutrients [16,17]. By way of example, creatine becomes conditionally essential in patients with chronic kidney disease as endogenous creatine production declines with the increasing degree of chronic kidney disease [18]. Similarly, exogenous creatine obtained from breast milk is essential during the first 12 months of life to support growth and maturation throughout this critical developmental period [19].

To establish Ubiquinol as a conditionally essential nutrient, integrated biochemical, clinical, and regulatory evidence is required, demonstrating that, under specific physiological or pathological conditions, endogenous Ubiquinol production is insufficient to maintain normal mitochondrial and antioxidant function and that dietary or supplemental Ubiquinol may be necessary to restore physiological homeostasis.

## 3. Endogenous Biosynthesis of Ubiquinol

Ubiquinol is the reduced form of CoQ10 that donates electrons and functions as a lipid-soluble antioxidant, whereas Ubiquinone must be reduced before it can served these roles [20]. Ubiquinol’s antioxidant activity is known to mitigate reactive oxygen species (ROS) propagation and lipid peroxidation central to iron-dependent cell death, i.e., ferroptosis, distinguishing it biochemically from the oxidized Ubiquinone form [21,22].

**Figure 1:**
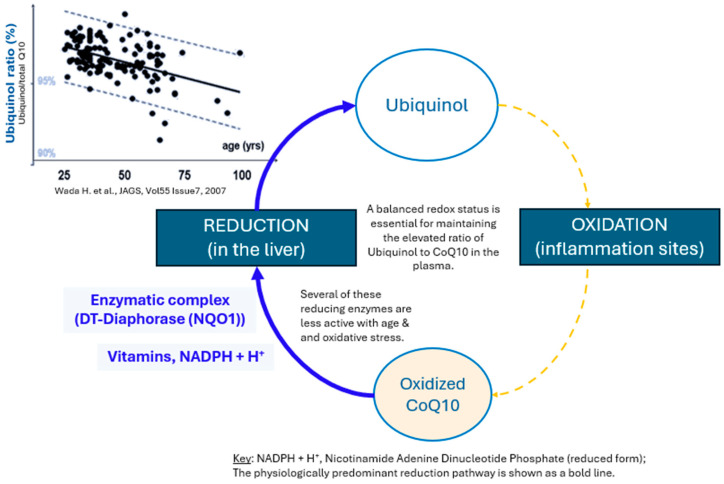
Major pathways involved in Ubiquinol metabolism. Source: Adapted from Kontush et al. (1999) [35,36].

Within cellular metabolism, CoQ10 interconverts between its reduced (Ubiquinol) and oxidized (Ubiquinone) forms [2], as shown in Figure 1. This predominantly occurs within cells in the mitochondria where CoQ10 shuttles electrons by cycling between oxidized (Ubiquinone) and reduced (Ubiquinol) states [2]. It is reduced by electron donation from Complex I (NADH–Ubiquinone oxidoreductase) or Complex II (succinate dehydrogenase) and then reoxidized back (Ubiquinol → Ubiquinone) in Complex III (“the Q cycle”), with Complex I and III being the preferential metabolic channels, rather than random membrane diffusion [23]. This helps to confer (1) enhanced catalytic efficiency, (2) metabolic flexibility, and (3) mitigation of ROS [23]. This cycle is central for oxidative phosphorylation and adenosine triphosphate (ATP) production [2]. Mitochondrial oxidoreductase enzymes also participate in this Ubiquinone–Ubiquinol redox cycle [2].

In the bloodstream, CoQ10 is predominantly transported in its reduced form, Ubiquinol, bound to low-density and very-low-density lipoproteins, regardless of whether it is initially consumed as Ubiquinone or Ubiquinol [24]. In human serum and biological tissues, more than 90% of CoQ10 also exists in the reduced Ubiquinol form [25]. During absorption, Ubiquinone is enzymatically reduced to Ubiquinol primarily within the lymphatic system [26]. Ubiquinol is largely responsible for antioxidant properties endogenously [20,27]. Through its antioxidant actions, it helps prevent the initiation and propagation of lipid peroxidation in biological membranes—a key mechanism that can lead to cellular damage, dysfunction, or death [20]. The antioxidant function of CoQ10 must be constantly regenerated from the oxidized Ubiquinone to its reduced Ubiquinol form [26]. CoQ10 biosynthesis requires a minimum of 15 genes [28]. Certain mutations in eight of these, i.e., PDSS1, PDSS2, COQ2, COQ4, COQ6, ADCK3, ADCK4, and COQ9, can result in CoQ10 deficiency, which may contribute to defective levels of OS and ATP production [28].

Infants and children with primary CoQ10 deficiency present with diverse clinical phenotypes, most often featuring developmental delay, myopathy, and multisystemic or tissue-specific manifestations of bioenergetic dysfunction [29], highlighting the critical importance of maintaining normal Ubiquinol homeostasis during this vulnerable developmental period. This, in turn, influences CoQ10requirements (and, consequently, Ubiquinol requirements), which are likely to be elevated [28]. Bacteria and yeast with mutations contributing to CoQ10 deficiency cannot grow if they are unable to respire, and in mammals, a lack of CoQ10 causes embryonic lethality [30].

Finally, while mitohormesis—a small, controlled amount of mitochondrial stress—may confer health benefits, increased endogenous or supplemental CoQ10may be advantageous under conditions of elevated OS [31]. Cells can increase or repair mitochondria in response to oxidative damage or higher energy needs, with redox signals activating transcription factors that remove damaged mitochondria or create new ones [32]. It is also important to consider that mitochondrial mass and quality are regulated tightly by mitobiogenesis (mitochondrial formation) and mitophagy (mitochondrial degradation), which are responsive to cellular energy needs and environmental cues [33], which appears to include Ubiquinol supply [2,34].

## 4. Dietary Sources

**Figure 2:**
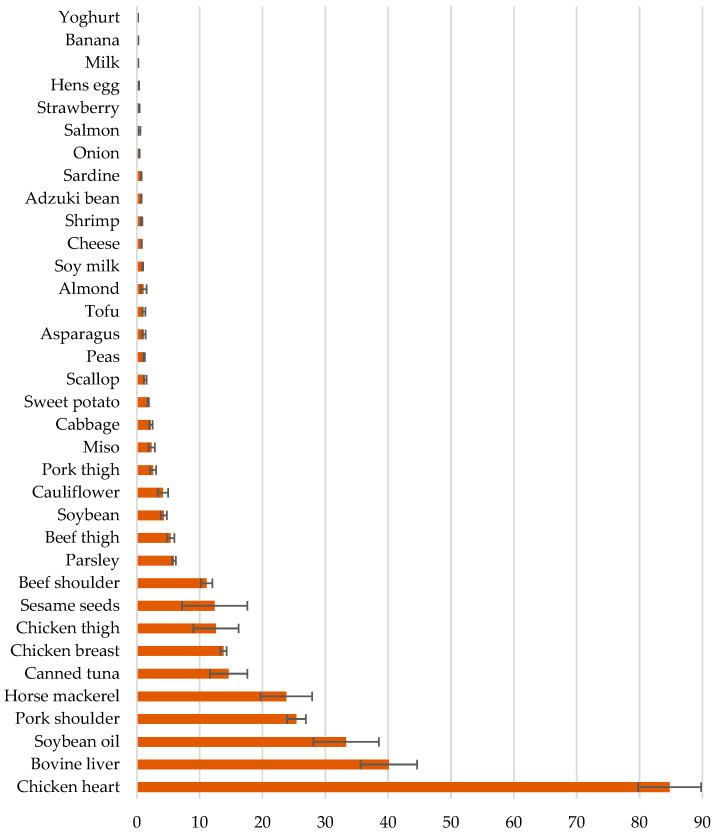
Different food sources providing Ubiquinol-10 (µg/g wet weight). Source: Data values extracted from Kubo et al. (2008) [38].

It is well recognized that some of the most abundant food sources of CoQ10 include meat, organs, fish, and vegetables [37]. Ubiquinol and Ubiquinone are found in food sources with Ubiquinol accounting for around 46% of total CoQ10 intake in some diets [38]. Certain organ meats such as bovine liver, chicken heart, and pork shoulder have a particularly high Ubiquinol content of more than 20 μg/g [38]. Ubiquinol has been found to be available in a range of foods including meat (2.63 to 84.8 μg/g), fish and shellfish (0.38 to 23.8 μg/g), vegetables (0.07 to 5.91 μg/g), pulses (0.72 to 4.3 μg/g), fruits (0.22 to 3.14 μg/g), potatoes (0.68 to 1.82 μg/g), and other foods such as eggs, dairy products, seeds, soybean oil, and miso, with levels ranging between and 0.18 and 33.3 μg/g in [38]. As shown in Figure 2, it can be observed that many foods providing some of the highest Ubiquinol levels are meat or fish derived, except for soybean oil and sesame seeds. If such foods are not consumed, then this may then impact on endogenous reserves. For example, the effects of a two-week diet without meat and poultry were studied among women (n = 22, 20–21 years) [39]. Average daily intakes of CoQ10 reduced from 2.1 to 1.1 mg/day, and the average serum reduced, oxidized, and total CoQ10 levels declined by 14%, 31%, and 16%, respectively, indicating that those following vegetarian and vegan of the EAT-Lancet Commission healthy reference diet may require other exogenous sources, such as supplements [39]. Since the dietary uptake of CoQ10 can be limited, the regulation of biosynthetic pathways becomes more important [40].

## 5. Life Stages Where Ubiquinol May Be Conditionally Essential

There are certain life stages during which Ubiquinol may become conditionally essential, including the reproductive years (for both male and female fertility), pregnancy and neonatal development, and advanced aging. Female oocytes rely heavily on mitochondria for energy [41], and CoQ10/Ubiquinol are critical for maintaining mitochondrial function and protecting against OS [42]. Male spermatozoa are also highly vulnerable to OS [43,44]. In the case of pregnancy, energetic requirements are increased [45]. Whilst a level of OS is a normal part of placental development, exaggerated OS may be associated with adverse pregnancy outcomes and can occur when antioxidant supply is limited [46]. With age, endogenous production of CoQ10/Ubiquinol declines (mitochondrial synthesis slows) [47]. This is particularly relevant given the increasing maternal and paternal ages at conception observed in many regions [48,49].

## 6. Ubiquinol and Male Fertility

Regarding reductive stress, there have been advancements in male infertility research over the last few decades [50]. Male infertility has been linked toOS, diminished antioxidant defenses, sperm DNA fragmentation, and impaired mitochondrial function within spermatozoa [51]. Around 30% to 80% of infertile men are reported to have elevated seminal ROS levels in the seminal fluid [52]. Case-control study findings have shown that lower total antioxidant capacity appears to induceOS, leading to sperm dysfunction and male infertility [53].

CoQ10 is particularly beneficial for sperm quality due to its antioxidant properties and is also a critical component of energy production [54]. One proposed mechanism by which CoQ10may improve sperm quality is by enhancing antioxidant enzyme activity, given that adequate ATP availability is critical for sperm production and motility [54,55]. CoQ10 is present in measurable concentrations in human semen, reflecting its essential roles in mitochondrial bioenergetics, cellular metabolism, and antioxidant defense mechanisms [56]. Given these roles in seminal fluid, the concentration of CoQ10 has been found to correlate to sperm count and motility [57].

**Figure 3:**
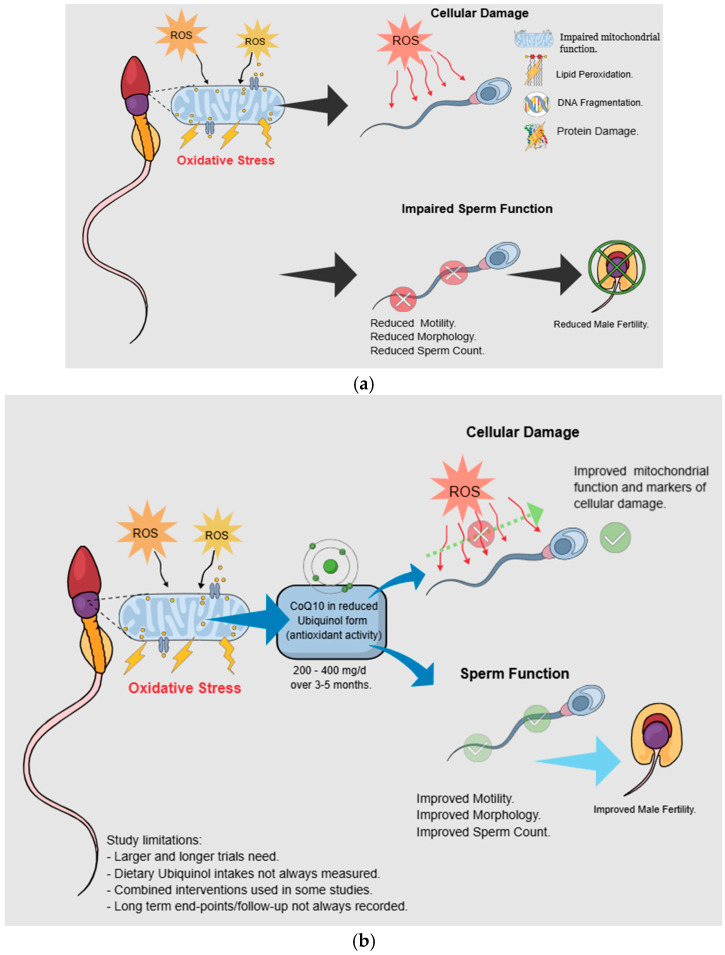
(**a**) Mitochondrial function, oxidative stress, and sperm attributes. (**b**) Ubiquinol, sperm attributes, and infertility management.

As shown in Table 1, several publications have investigated the role(s) of CoQ10 in the reduced Ubiquinol form in the context of male infertility [58,59,60,61,62,63,64]. Potential mechanisms of action and illustrative findings from these studies are shown in Figure 3a,b. Most recently, a trial administering 200 mg Ubiquinol (alongside D-aspartic acid and zinc) to 24 infertile male patients showed significant improvements in sperm motility after 3 months, although it should be considered that this was a combined intervention; thus, individual Ubiquinol effects are difficult to determine [63]. Similar results were observed in a randomized, double-blind, placebo-controlled interventional study of normospermic and oligospermic men, where co-supplementation with Ubiquinol and creatine for eight weeks led to significant improvements in several sperm quality biomarkers, although this was a pilot study with a small size and short study duration [64].

Several other studies have focused on idiopathic oligoasthenospermia—a condition characterized by reduced sperm concentration and motility [58,59,60,62]. In a short-term 3-month prospective study, men with received either 200 mg/day (n = 35) or 400 mg/day (n = 30) of CoQ10 in its reduced form Ubiquinol [59]. Both levels of Ubiquinol significantly increased total antioxidant capacity, sperm concentration, and both progressive and total motility compared to baseline [59]. It should be considered, however, that dietary Ubiquinol intakes and longer-term measures such as pregnancy and live birth rates were not measured [59]. In a larger and 3-month prospective controlled study with a similar study population, 50 men and 50 fertile controls were given 200 mg of Ubiquinol daily [60]. Among the infertile men, Ubiquinol supplementation significantly improved antioxidant status and sperm quality while also reducing ROS and sperm DNA fragmentation [60].

A significantly larger study involving 178 infertile men and 84 fertile controls found that daily supplementation with 200 mg of Ubiquinol over six months led to improved antioxidant markers, reduced sperm DNA fragmentation, and a pregnancy rate of 24.2% [58]. Although this represents a significant improvement over baseline, the pregnancy rate remained lower than in the control group (95.2%), suggesting that while Ubiquinol offers meaningful improvement of male fertility, it may not fully address underlying issues such as advanced paternal age or severe sperm abnormalities [58]. Earlier research conducted on 228 men with unexplained infertility found that 200 mg Ubiquinol administered daily over 26 weeks appeared to improve sperm density, motility, and morphology, although functional measures such as sperm DNA fragmentation were not measured [61]. Other research administering 300 mg/day Ubiquinol once daily to male patients (n = 50) with idiopathic oligoasthenospermia observed significant improvements in sperm motility, total motility, and antioxidant biomarkers compared to baseline [62]. It should, however, be considered that participants were recruited from a single region, which may limit the generalizability of the results [62]. Additionally, dietary intake of Ubiquinol was not measured, which would have been a useful addition [62].

**Table 1:** Key studies focusing on Ubiquinol and male fertility.

Author (Year)	Study Design	Patients/Controls	Intervention and Dose	Parameters Assessed	Outcome
Gamal El Din S.F. et al. (2025) [63]	3-month DB PC RS.	N = 75 infertile patients.	200 mg/d Ubiquinol + 2660 mg/d d-aspartic acid + 10 mg zinc (n = 24). Placebo (starch granules) (n = 24).	Semen parameters	The active intervention showed sig. improvements in progressive sperm motility after 3 months.
Nedeljkovic D. et al. (2025) [64]	8-week randomized controlled pilot trial.	n = 15 including n = 5 oligospermic patients.	200 mg/d Ubiquinol + 5 g/d creatine monohydrate plus.	Sperm quality biomarkers.	Oligospermic men receiving creatine-plus-Ubiquinol had improved sperm concentration at follow-up.
Alahmar A.T. et al. (2022a) [58]	6-month prospective controlled clinical study.	n = 178 with idiopathic OA. n = 84 fertile men (controls).	200 mg/d of CoQ10 as Ubiquinol.	Semen parameters. Time to pregnancy.	Ubiquinol therapy sig. improved semen parameters (sperm concentration and motility) antioxidant measures and reduced SDF.
Alahmar A.T. et al. (2022b) [62]	3-month prospective controlled study.	n = 50 with idiopathic OA. n = 35 fertile controls.	300 mg/d oral CoQ10 as Ubiquinol.	Sperm motility. Antioxidant status.	The intervention resulted in sig. ↑ sperm progressive motility, total motility, seminal TAC, SOD, GPx, and ↓ ROS compared to baseline.
Alahmar A.T. et al. (2021) [60]	3-month prospective controlled study.	n = 50 with OAT n= 50 fertile men (controls).	200 mg/d of oral CoQ10 as Ubiquinol.	Sperm DNA damage.	Ubiquinol administration to men with idiopathic OAT sig. improved sperm quality, antioxidant status and ↓ ROS and SDF levels.
Alahmar A.T. et al. (2019) [59]	3-month prospective controlled clinical study.	n = 65 patients with idiopathic OAT received.	200 mg/d (n = 35) 400 mg/d (n = 30) of CoQ10 as Ubiquinol.	Semen parameters. Antioxidant status.	Ubiquinol improved semen parameters (sperm concentration, progressive and total motility) and antioxidant status with a greater improvement in those taking 400 mg/d.
Safarinejad M.R. et al. (2012) [61]	26-week DB PC RS.	n = 228 men with unexplained infertility.	200 mg/d Ubiquinol or placebo.	Semen parameters.	Ubiquinol sig. improved sperm density, sperm motility, and sperm morphology.

Key: DB, double-blind; DNA, deoxyribonucleic acid; SDF, sperm DNA fragmentation; GPx, glutathione peroxidase; sig., significantly; OA, idiopathic oligoasthenospermia; OAT, oligoasthenoteratozoospermia; PC, placebo-controlled; RS, randomized study; ROS, reactive oxygen species; SOD, superoxide dismutase; TAC, total antioxidant capacity; “↑” increased and “↓” reduced.

## 7. Ubiquinol and Female Fertility

Female fertility is influenced by a wide range of factors, with oocyte quality and ovarian reserve representing major limiting determinants [65]. Other factors that can influence female fertility can include chronic and systemic diseases, infections, alcohol, caffeine, nutrition, weight, exercise, smoking, illicit drug use, psychological stress, and age at which to start a family along with environmental and occupational exposures [66].

Even before conception, research shows that hormonal contraception use reduces plasma CoQ10 and serum total antioxidant capacity levels, indicating an increased risk of OS [67]. Furthermore, an open-label pilot study in 86 premenopausal healthcare workers (21–48 years) found that daily Ubiquinol supplementation (150 mg/day) from the fourth to seventh menstrual cycle reduced menstrual symptoms, including premenstrual irritability, and improved sleep quality, with more pronounced effects in participants aged ≥36 years [68]. However, it should be considered that this was a pilot study with limited statistical power, with a lack of randomization and control [68].

Several studies have examined the effects of Ubiquinol supplementation during fertility treatment (Table 2) [69,70,71]. In one trial, women (n = 135) with unexplained infertility undergoing IVF took 100 mg CoQ10 and 600 mg omega-3 daily for 2 months before undergoing ovarian stimulation (n = 164 acted as controls) by gonadotropin [69]. Clinical pregnancy rate was significantly higher amongst those using the supplements, and the dose of gonadotropin required for stimulation was also lower in patients using antioxidants, possibly due to CoQ10 acting as an antioxidant and agent that increases mitochondrial energy production [69]. It should, however, be considered that combined supplementation was used in this trial, so specific effects of Ubiquinol per se are more challenging to identify [69]. There was also no randomization, which may have resulted in selection bias [69]. In another study of 75 patients, daily Ubiquinol (with dehydroepiandrosterone (DHEA) and triiodothyronine (T3) over 8 weeks enhanced mitochondrial energy metabolism by prioritizing the tricarboxylic acid (TCA) cycle over glycolysis and inhibiting ferroptosis, although it should again be considered that this was a multi-ingredient intervention [70]. Furthermore, in women with polycystic ovary syndrome (PCOS) and Clomiphene Citrate (CC) resistance (n = 148), Ubiquinol (100 mg/day) combined with CC improved ovarian responsiveness, achieving outcomes comparable to human menopausal gonadotropin (hMG) therapy [71]. Once again, this was a combined treatment intervention, but Ubiquinol may act as an adjunctive, potentially enhancing ovarian function and fertility outcomes, particularly in CC-resistant patients [71]. Continued research using randomized methods, longer-term fertility outcomes, and controlled Ubiquinol interventions are needed.

Amongst women, mitochondrial DNA (mtDNA) mutations accrue in oocytes with age, compromising oocyte functionality [72], with animal studies clearly demonstrating this [73]. Some evidence from animal studies suggests that Ubiquinol plays a role in rescuing oocyte quality, beyond the effects of OS alone [74,75]. For example, research using porcine oocytes found that Ubiquinol-10 administration promoted mitochondrial renewal by upregulating mitochondrial biogenesis proteins SIRT1 and PGC-1α and enhancing mitophagy via PINK1 and PARKIN, which together increased the number of active mitochondria and ATP production, improving oocyte quality beyond its antioxidant effects [74]. Ubiquinol administration also improved mitochondrial physiology central to oocyte function in an aging murine model [75].

**Table 2:** Key studies focusing on Ubiquinol and female fertility.

Author (Year)	Study Design	Patients/Controls	Intervention and Dose	Parameters Assessed	Outcome
Kinoshita T. et al. (2025) [68]	7 menstrual cycles—open-label pilot study.	n = 86 healthcare workers 21–48 yrs.	150 mg/d Ubiquinol as two soft 75 mg capsules.	Menstrual symptoms.	In those aged ≥36 ys, Ubiquinol may reduce premenstrual irritability and improve sleep quality.
Lin P.H. et al. (2023) [70]	8-week supplement intervention before undergoing IVF.	n = 75 with ovarian senescence.	Ubiquinol CoQ10 Capsules (dose not specified), DHEA capsules, and Cleo-20 triiodothyronine soft capsules.	Ovarian aging.	The supplement regimen containing Ubiquinol was associated with improved mitochondrial energy-metabolism markers in ovarian cells.
Ammar I.M.M. et al. (2021) [71]	RCT.	n = 148 women with PCOS and Clomiphene Citrate resistance.	100 mg/d Ubiquinol starting on cycle day 2 and continued until the day of hCG triggering.	Ovulation induction.	Ubiquinol addition to Clomiphene Citrate improved ovarian responsiveness in Clomiphene-Citrate-resistant patients when compared to conventional hMG stimulation.
Ozdemir A. et al. (2019) [69]	8-week supplementation intervention before ovarian stimulation.	n = 299 patients undergoing IVF-ICSI for unexplained infertility.	100 mg/d Ubiquinol + omega-3 fatty acids (300 mg/d EPA and 230 mg/d DHA) (n = 135). No supplement control (n = 164).	Pregnancy rate.	The pregnancy rate of patients using Ubiquinol was sig. higher than those not using the supplement.

Key: DHA, docosahexaenoic acid; DHEA, dehydroepiandrosterone; EPA, eicosapentaenoic acid; hCG, human chorionic gonadotropin; ICSI, Intracytoplasmic Sperm Injection; IVF, in vitro fertilization; PCOS, polycystic ovary syndrome; RCT, randomized controlled trial.

## 8. Advanced Paternal and Maternal Aging

With the increasing trend of delayed parenthood, there are important implications for reproductive function [49,76]. The total body content of CoQ10 is only estimated to be 500–1500 mg and recedes with age [77]. Women tend to have lower cholesterol-adjusted CoQ10 levels compared to men [78]. Previous research shows that CoQ10 synthesis within the human body begins to decline at around 20 years of age [79].

In men, aging is associated with a decline in antioxidant defense mechanisms, leading to the accumulation of ROS in spermatozoa, which impairs their structural integrity and function [76]. For instance, paternal age in the United Kingdom has increased from 30.7 years in 1990 to 33.4 years in 2017, while in Italy, it has risen from 34.2 to 35.5 years between 2000 and 2018 [49]. Similarly, women experience a progressive decline in oocyte quality and quantity with age, attributed in part to OS and mitochondrial dysfunction [80]. Ovarian aging, characterized by the gradual loss of ovarian function, is influenced by mitochondrial dysfunction, which contributes to energy deficits, DNA damage, and increasedOS, all of which compromise normal ovarian physiology [81]. The live birth rate for women under 35 is approximately 43% but decreases sharply to 6% in women over 42.5 years, primarily due to diminished ovarian reserve, reflecting declines in oocyte quality and quantity. The substantial improvement in live birth rates with the use of donated oocytes in older women underscores the critical role of oocyte competence in successful conception [82].

Across the European Union, approximately 1 in 20 births are to women aged 40 years or older, with higher proportions observed in Ireland, Spain, and Italy [48]. In vitro research has shown that postovulatory oocytes, cultured for 48 h with Ubiquinol, were protected fromOS, and the number of active mitochondria and ATP level were also significantly increased [74]. These findings suggest that Ubiquinol supports mitochondrial renewal and quality by promoting mitochondrial biogenesis and enhancing mitophagy, the selective removal of damaged mitochondria [74].

In other research involving 70 infertile women with aging ovaries, participants were randomized to receive either a combination of 30 mg Ubiquinol, 25 mg DHEA, and 300 mg T3 or no treatment for eight weeks. Ferredoxin 1 (FDX1), an iron–sulfur protein essential for mitochondrial electron transport, was used as a biomarker of ovarian aging. Ubiquinol supplementation significantly increased FDX1 gene expression, suggesting a role in improving oocyte quality in aging women [83]. It was found that Ubiquinol supplementation increased FDX1 gene expression, indicating that Ubiquinol helps to reinforce oocyte quality improvement in aging women [83]. Additionally, a cross-sectional study of 32 women with premature ovarian insufficiency (POI) versus 58 age-matched controls found an inverse correlation between serum CoQ10 levels and POI, suggesting antioxidant deficiency as a risk factor, while Ubiquinol supplementation has been shown to improve oocyte quality and ART outcomes, including IVF and ICSI, particularly in women of advanced reproductive age [84].

## 9. Pregnancy and Early Life

Pregnancy is a complex and physiologically demanding life stage [45]. Indeed, the body needs to adapt to increasing energetic requirements due to both embryo and fetal development and failure to adapt could have health ramifications for the mother and child [45]. Mitochondria are the predominant organelle responsible for energy generation in the form of ATP, thus their function becomes particularly important across this life stage [45]. It is also now becoming more widely recognized that OSis heightened during pregnancy, and amplified states of OS can trigger certain detrimental outcomes such as pre-eclampsia, pregnancy loss, embryonic resorption, intrauterine growth restriction, and fetal developmental abnormalities [85].

Several studies have reported changes in maternal CoQ10 status during gestation, implying physiological adaptations to meet the increased energy demands and levels of OS in pregnancy [86,87,88,89]. For example, a longitudinal study with 50 healthy pregnant women (mean age 31 years) showed that CoQ10 levels increased from the first to the third trimester, with levels in the third trimester being significantly positively associated with infant body weight [86]. These findings highlight that the rise in maternal CoQ10 likely reflects adaptive regulation of endogenous biosynthesis to support higher mitochondrial activity in maternal and placental tissues and to counterbalance increased OS during the last half of normal pregnancy. Other research similarly reports that CoQ10 typically rises during each trimester but that spontaneous contractile activity (spontaneous abortion) is associated with low CoQ10 levels [87].

Further research on amniotic CoQ10 levels [88] shows that in cases of fetal growth restriction, amniotic CoQ10 levels were higher compared to healthy pregnancies. This suggests that increased CoQ10 may be a compensatory response to elevated OS in such conditions [88]. Other research has shown that CoQ10 supplementation (200 mg from 20-week gestation until delivery) could lower the risk of developing pre-eclampsia amongst women at risk of developing the condition [89]. Animal models using Wistar rats suggest that CoQ10 can enhance the function of mitochondria in the placenta [90]. This appeared to attenuate pre-eclampsia symptoms—the weight and number of pups in the pre-eclampsia CoQ10 group were significantly larger than the distilled water control group [90]. Other animal models have demonstrated that CoQ10 supplementation can improve the reproductive performance and embryonic survival of high parity sows, possibly by improving the development of uterine function [91].

Research specifically investigating Ubiquinol-10 has shown that, while mothers have higher total venous CoQ10 levels (Ubiquinol-10 + Ubiquinone-10) compared to umbilical cord blood, fetuses exhibit significantly higher levels of oxidized CoQ10 [92]. This is reflected in a higher Ubiquinone-10 to total CoQ10 ratio, indicating greater OS in the fetal environment [92]. These findings suggest that, due to both its antioxidant properties and low levels in the fetus, Ubiquinol may be a key candidate as a conditionally essential nutrient during development. The fetus may potentially benefit from better antioxidant defense—either through endogenous production or maternal support (e.g., from diet or supplementation). Further research has linked heightened OS and defective mitochondrial function as shared features of brain/neurodevelopmental conditions with CoQ10 Ubiquinol (alongside B-vitamin and vitamin E) potentially helping to cognitive functioning, especially in the presence of intellectual disability [93].

## 10. Bioavailability and Safety

Studies have investigated the bioavailability of Ubiquinol in supplemental form [94,95]. Ubiquinol is well absorbed from the gut, and daily doses of up to 300 mg/day over 4 weeks have been found to be well tolerated [96]. Some evidence suggests that Ubiquinol may have enhanced bioaccessibility and bioavailability compared to Ubiquinone [94,95,97]. Findings from a 4-week trial show that 200 mg/d of Ubiquinol was better absorbed and 70% more bioavailable than Ubiquinone [94]. This could be due to Ubiquinol being more efficiently incorporated into mixed micelles during small intestinal digestion and having better trans-epithelial transport [95]. Ubiquinol (when compared with Ubiquinone-10) also appears to increase levels of CoQ10 in mitochondria and reduce oxidative damage in the brainstem of mice [98]. Other murine models have shown that both Ubiquinol and Ubiquinone are well absorbed by small intestinal tissue, mostly in its original form [99].

Research shows that both 100 mg and 150 mg Ubiquinol taken daily over 4 weeks could help to relieve mild fatigue in healthy adults with no side effects reported [100]. One trial evaluating the safety of Ubiquinol found that doses up to 300 mg/day were safe and bioavailable, with steady-state levels being ready within 2 weeks of supplementation [96]. Other science has shown that single or multiple doses of Ubiquinol (up to 300 mg) was well absorbed from the gastrointestinal tract, and no safety concerns were raised during the 4-week trial or in the 2 weeks after treatment completion [96]. A recent phase 2 trial also found that 1500 mg/day of Ubiquinol was well tolerated when taken in the longer term over 48-weeks by patients with multiple system atrophy (progressive neurodegenerative disease) [101].

## 11. Discussion

**Figure 4:**
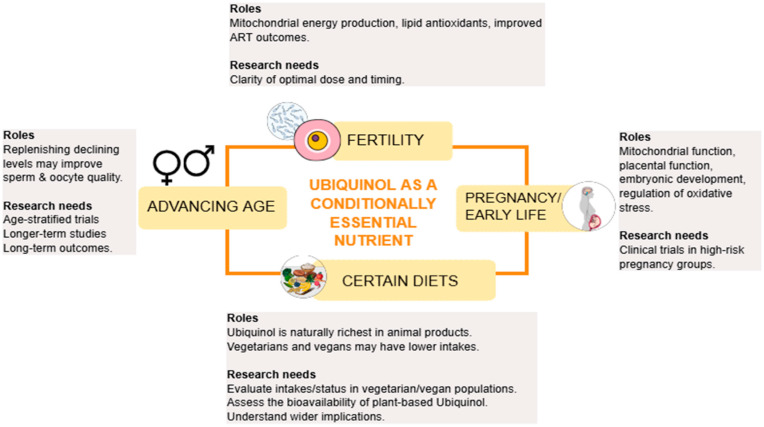
Ubiquinol as an essential nutrient for reproduction: roles and research needs. Key: ART, assisted reproductive technologies.

Ubiquinol is not officially an essential nutrient for the lay general population as we can produce it endogenously. However, emerging studies suggest that, under certain conditions, the reduced form of CoQ10—Ubiquinol—may meet the criteria to be considered a conditionally essential nutrient. This may be underpinned by a number of its important physiological roles. The main roles and research needs in relation to Ubiquinol as a conditionally essential nutrient are displayed in Figure 4.

Firstly, Ubiquinol plays a central role in mitochondrial ATP production [2]. This is central to energy-demanding reproductive processes, which include providing sufficient energy for preimplantation embryo development [102], ovarian aging, and associated oxidative damage [103], and for sperm motility and function [104,105]. As we have seen in the present narrative review, inadequate Ubiquinol levels may impair mitochondrial efficiency and contribute to reduced gamete quality [2,83,106]. Secondly, Ubiquinol has an important role as a lipid-soluble antioxidant that protects cells from OS, including oocytes and sperm cells [9,107,108]. Ubiquinol plays an important role in helping to protect against oxidative damage to mitochondrial protein and DNA during states of lipid peroxidation [109]. It also neutralizes lipid peroxides and helps to regenerate vitamin E—mechanisms that may help to maintain gamete integrity [20,110]. Thirdly, it is well recognized that CoQ10 synthesis declines with age, possibly due to reduced expression of genes for synthesis, particularly after the mid-30s in some tissues [75,78]. This coincides with a drop in fertility in both males and females, which may be attributed to mitochondrial decline and heightened OS [103,111,112,113]. Given that many couples are waiting until later to have children in life and supplementation restores Ubiquinol levels, this makes Ubiquinol a conditionally essential nutrient amongst those of older reproductive age.

In pregnancy and early life, embryo development relies on suitable mitochondrial activity and protection against OS [45,92]. Ubiquinol is also important as it helps to provide an adequate energy supply for cell division, implantation, and placental development [2,45]. Animal models have shown that Ubiquinol helps to improve mitochondrial renewal, thus improving oocyte aging [74]. Similarly, human trials have shown that Ubiquinol CoQ10 as part of a wider formulation improved IVF outcomes in aging cells, potentially by improving energy metabolism and reinforcing oocyte quality in older women [70]. Murine models have also shown that Ubiquinol-cytochrome c reductase core protein I (UQCRC1; a key component in the respiratory chain) appear to play a critical role in embryo survival [114]. Amongst males, Ubiquinol supplementation has been associated with improved sperm morphology, motility, concentration, reduced DNA fragmentation, OS, and improved chances of conception [12,56,60,115]. Deficiencies or insufficiencies due to dietary shortfalls or genetic differences could impact on reproductive success, thus making it “conditionally essential” in these contexts.

## 12. Future Frontiers

Future research on Ubiquinol should aim to move beyond the current focus on short-term supplementation trials and toward a more comprehensive understanding of its role across the human lifespan. While current evidence supports benefits in reproductive health and fertility, there remains a major knowledge gap regarding Ubiquinol requirements during early developmental stages—such as infancy, childhood, and adolescence—when mitochondrial activity, oxidative metabolism, and tissue growth are particularly high. Establishing age-specific reference ranges for circulating Ubiquinol and corresponding dietary or supplemental needs represents an important frontier.

Equally, periconceptional, pregnancy, and lactation periods warrant deeper exploration, as maternal Ubiquinol status likely influences fetal and neonatal mitochondrial function and antioxidant capacity. Pharmacokinetic studies across these life stages are needed to determine absorption, bioavailability, and tissue distribution, which may differ markedly from adult populations. Finally, the integration of nutrigenomic and metabolomic profiling could clarify interindividual differences in Ubiquinol metabolism and guide personalized recommendations. Defining these parameters will be essential to establish evidence-based dietary reference intakes and optimize Ubiquinol’s use as a conditionally essential nutrient across different age and physiological stages.

Overall, the following can be concluded:

- Ubiquinol is an important lipid-soluble antioxidant that is critical to mitochondria function [2,3].
- There are not yet official intake recommendations for Ubiquinol, but some of the most predominant food sources include meat, fish, eggs, and dairy [38]. Consequently, individuals following plant-based diets or restricting these food groups may be at risk of lower habitual intakes, potentially resulting in reduced Ubiquinol consumption.
- It has been advised that to reduce sperm damage and improve sperm motility, count and morphology 200 mg CoQ10 (as Ubiquinol) could be taken daily, increasing to 400 mg per day for those with serious difficulties [54]; however, consensus is lacking across scientific domains regarding the optimal dose and duration of Ubiquinol therapy.
- It would be useful to obtain more data on “long-term” Ubiquinol intakes using food frequency questionnaires across a range of European regions to help build a reliable picture of habitual Ubiquinol intakes.
- Greater efforts are needed to educate healthcare professionals and consumers about the importance of Ubiquinol, how it can be obtained, and the life stages during which its supply is most critical.
- If Ubiquinol is not obtained in the levels needed from dietary sources per se, then supplementation strategies may be required, especially at certain key life stages such as conception, pregnancy, and advanced maternal/paternal age. Food supplements can play a useful role in helping to offset gaps, helping to improve fertility-related wellbeing [116].

## 13. Conclusions

In conclusion, Ubiquinol may become essential under certain physiological or pathological conditions. Pre-conception use, particularly in the presence of fertility challenges, advancing maternal or paternal age, and increased demands during the reproductive years are examples of life stages when Ubiquinol requirements may be elevated. There is now a need for more targeted research and the development of official intake recommendations. Ongoing research is needed to determine habitual Ubiquinol intakes and formulate benchmark levels of recommended intakes. Research communities and public authorities should work together to help raise awareness of this important compound that appears to have valuable roles across the periconceptual and reproductive life stages.

## Data Availability

No new data were created or analyzed in this study.

## Abbreviations

The following abbreviations are used in this manuscript:

ART	Assisted Reproductive Technology
ATP	Adenosine Triphosphate
CC	Clomiphene Citrate
CoQ10	Coenzyme Q10
DHEA	Dehydroepiandrosterone
FDX1	Ferredoxin 1
hMG	Human Menopausal Gonadotropin
IVF	In Vitro Fertilization
NADH	Nicotinamide Adenine Dinucleotide (Hydrogen)
PCOS	Polycystic Ovary Syndrome
POI	Premature Ovarian Insufficiency
ROS	Reactive Oxygen Species
TCA	Tricarboxylic Acid Cycle
T3	Triiodothyronine
UQCRC1	Ubiquinol-Cytochrome c Reductase Core Protein I
